# Comparative Longitudinal Analysis of Malignant Transformation in Pleomorphic Adenoma and Recurrent Pleomorphic Adenoma

**DOI:** 10.3390/jcm11071808

**Published:** 2022-03-25

**Authors:** Sung Yong Choi, Jaehyuck Choi, Inwoo Hwang, Junhun Cho, Young-Hyeh Ko, Han-Sin Jeong

**Affiliations:** 1 Department of Otorhinolaryngology-Head and Neck Surgery, Samsung Medical Center, Sungkyunkwan University School of Medicine, Seoul 06351, Korea; sungyong82.choi@gmail.com (S.Y.C.); jh1.choi@samsung.com (J.C.); 2 Department of Otorhinolaryngology-Head and Neck Surgery, Uijeongbu Eulji Medical Center, Eulji University School of Medicine, Daejeon 34824, Korea; 3 Department of Pathology, Samsung Medical Center, Sungkyunkwan University School of Medicine, Seoul 06351, Korea; inwoo87.hwang@samsung.com (I.H.); junhun.cho@samsung.com (J.C.); yhko310@skku.edu (Y.-H.K.)

**Keywords:** salivary glands, salivary gland neoplasms, pleomorphic adenomas, local neoplasm recurrence, neoplastic cell transformation

## Abstract

Background: Recurrence in pleomorphic adenoma (PA) has been debated as a risk factor for malignant transformation (MT). In this study, we investigated whether recurrence is a risk factor for MT, by longitudinally analyzing cases with recurrent PA (RPA), and carcinomas from PA (CXPA) or RPA (CXRPA). Methods: The study population included 24 CXPA, 24 RPA, 6 CXRPA, and 386 PA cases (study period 2010–2018). Time and event data were collected from the medical documents to identify the time–event sequences. Results: The time interval to MT in CXRPA was significant longer than that of benign recurrence (median 342.0 vs. 109.5 months). In CXRPA, the recurrence intervals were not shorter than those in RPA according to recurrence frequency. Crudely, the MT rate was 5.9% among primary cases and 20.0% among recurrent cases. However, the time-adjusted MT rates increased up to 11.4% (incubation time > 60 months) and 20.0% (>120 months) in primary cases, which were not different from recurrent cases. Conclusion: In these longitudinal analyses, we did not find any clinical evidence that recurrence facilitates MT in the background of PA. Instead, a long incubation time seems to be a key factor for MT of underlying RPA.

## 1. Introduction

Malignant transformation from preexisting benign pleomorphic adenoma (Carcinoma ex pleomorphic adenoma, CXPA) occurs in less than 5% of cases with pleomorphic adenoma (PA) of the salivary gland [1,2,3]. Malignant transformation is a consequence of the accumulation of genomic changes over time to gain malignant potential [4]. Therefore, surgery is recommended for all PAs at the time of diagnosis. Surgery cures more than 95% of PAs of the salivary gland, and 2–7% of cases recur [5,6]. Several major risk factors for recurrent PA (RPA) include surgery-related factors such as violation of the tumor capsule, tumor rupture and incomplete excision [7]. Others have also described incomplete or uneven tumor capsulation, pseudopodia, and satellite nodule as additional risk factors for recurrence [8,9]. These pathologic features can also contribute to incomplete excision or result in microscopic residual disease in the surgical field.

Interestingly, recurrence (itself) in PA is considered a risk factor for malignant transformation of RPA (Carcinoma ex recurrent pleomorphic adenoma, CXRPA) in some studies analyzing RPA [10,11,12,13]. However, how repeated recurrences or surgeries promote the occurrence of CXRPA remains unclear [4]. The wound healing process or microenvironmental changes may influence the malignant transformation of RPA; however, this has not been demonstrated [13]. A recent study indicated that malignant transformation rates were not different between the first and second recurrences (6.2–6.7%) [14]. They also suggested a more conservative approach for RPA, because the risk of malignant transformation was low, but the risk of facial nerve injury was high in RPA [14].

In this paper, we attempt to answer whether recurrence (itself) is a risk factor for CXRPA. To attain this end, we longitudinally analyzed cases of RPA, CXPA or CXRPA, to capture the time–event sequence in a disease-spectrum of PA and corresponding CXPA. Throughout this study, we could only provide postulation for the relationship between recurrence and formation of CXRPA, because of the limited number of CXRPA patients. Nevertheless, our suggestion that recurrence itself is not a risk factor for CXRPA will provoke valuable discussion in this field.

## 2. Materials and Methods

CXRPA is not an accurate term for pathological diagnosis. In this paper, we defined CXRPA as a malignant tumor arising from recurrent PA. Therefore, the exact pathological diagnosis (category) is CXPA. Recurrence was defined as a clinical, radiological or pathological diagnosis of PA after a previous surgery in the same gland, with a disease-free interval. Meanwhile, residual disease indicated a case having persistent clinical disease without a disease-free interval after incomplete surgery (tumor cell-positive at the resection margin).

### 2.1. Study Subjects

This study was a retrospective analysis using clinical and pathological data from patients with PA, RPA, CXPA, and CXRPA. The study protocol was approved by our Institutional Review Board. The clinical data used in this study were de-identified. Based on an electronic search of our salivary gland tumor registry, we extracted cases diagnosed as PA, RPA, CXPA, and CXRPA. The study period spanned from 2010 to 2018, and included consecutive cases during that period. We did not set up a study hypothesis for this retrospective study initially because cases were rare and the study mainly presented descriptive findings.

The study population included 24 CXPA patients, 24 RPA patients (28 recurrences) and 6 patients with carcinomas from RPA (CXRPA) (total 13 recurrences, 10 recurrences for malignancy). We did not limit the primary sites to the major salivary glands, and instead included other sites of the head and neck. However, most tumors arose from the parotid gland, except one CXRPA in the lacrimal gland. Additionally, we enrolled 386 cases with PA, which were diagnosed during the same period (2010–2018) for comparison.

### 2.2. Collection of Clinical Data

We applied the following inclusion criteria: (i) pathologic verification of CXPA and CXRPA with background PA or a history of previous PA according to the World Health Organization (WHO) classification of salivary gland malignancies [15]; (ii) more than 36 months of follow-up after the end of treatment. 

We retrospectively collected the following clinical characteristics: age, gender, preoperative work-ups, tumor-node-metastasis (TNM) staging (American Joint Committee on Cancer staging manual, 8th edition) in cases of malignancy [16], treatment modalities and outcomes. To identify the time–event sequences in cases of recurrence or malignant transformation, we focused on the time point of each event in individual cases. Time and event data were collected from medical documents or patient histories.

### 2.3. Pathological Analyses

To confirm diagnoses, surgical or biopsy specimens were reviewed by two pathologists (J. Cho, Y.-H. Ko) with more than 5 years of experience in salivary gland pathology. Any ambiguity in the diagnosis was resolved by a joint discussion. Histological typing was performed or revised according to the 2017 World Health Organization classification of salivary tumors [15]. The histological tumor grade was defined as low, intermediate or high according to the cytological features and histomorphologic architectures [17,18]. 

Benign PAs were further classified into four subtypes: type 1: 30–50% of myxoid stroma; type 2: >80% of myxoid stroma; type 3: <30% of myxoid stroma and cells >80%; and type 4: <30% of myxoid stroma and uniformly differentiated epithelial cells [19,20]. Among 386 PA cases, we selected 30 consecutive cases that were diagnosed and treated in 2018, because of easy accessibility. We subclassified 30 PA and 24 RPA cases according to the above criteria.

### 2.4. Statistical Analyses

The results are presented mainly in a descriptive manner (numbers, frequencies, medians and interquartile ranges). The non-parametric Mann–Whitney U test was used to compare the time-to-event duration among disease categories. For cases with statistical analysis, a *p*-value < 0.05 was considered significant. Statistical analyses were performed using the Statistical Package for the Social Sciences software version 20 (IBM Corporation, Armonk, NY, USA).

## 3. Results

### 3.1. Subject Characteristics

**Figure 1:**
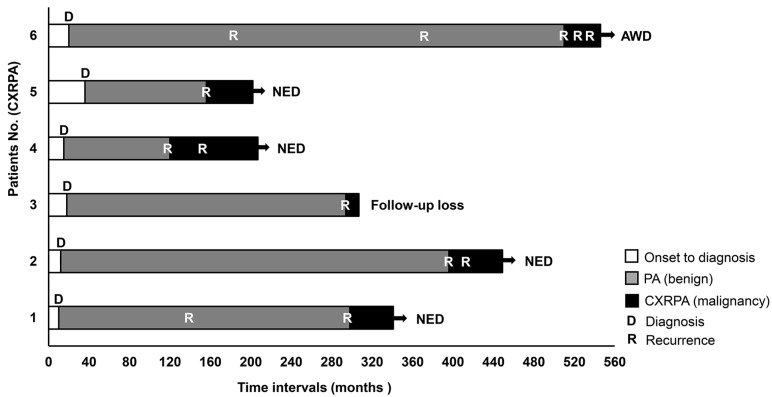
Clinical courses in patients with carcinoma ex recurrent pleomorphic adenoma. AWD, Alive with disease; NED, no evidence of disease.

The baseline characteristics of the enrolled patients are presented in Table 1. The median age of patients with RPA was lower than that of PA patients (*p* = 0.044). Meanwhile, the age distribution of CXPA and CXRPA patients was similar. In benign tumors, there was a female predominance. In contrast, male sex was dominant among malignant cases. In addition to patient information, we also analyzed the tumor characteristics of each surgical procedure. In patients with CXPA (*n* = 24), we performed upfront surgery in 21 patients and three had systemic chemotherapy for metastatic disease without surgery. In RPA (*n* = 24), 21 patients had one recurrence in their clinical courses (time intervals: median 113.0 months, interquartile range (90.0–186.0)), while two had two recurrences (time intervals: median 53.0 months, interquartile range (32.0–75.3)) and one had three recurrences (time intervals: median 69.0 months, interquartile range (44.0–102.5)). Therefore, 28 surgeries were conducted for RPA. Six patients had carcinoma that arose from PA recurrence (CXRPA) (Figure 1). For those, we performed 13 salvage surgeries including three for benign recurrences and 10 for malignant diseases.

The tumors were smaller in size and greater in number in RPA cases than in primary PA cases. The TNM staging of malignant disease was not significantly different between CXPA and CXRPA cases. Most malignant tumors were localized without metastasis (79.1% of CXPA, 60.0% of CXRPA). Wide resection of tumors and postoperative radiation was a major treatment for malignant diseases. As expected, the occurrence of postoperative facial weakness (more than 6 months after surgery) increased to over 25.0% in revision surgery cases.

### 3.2. Pathology Analyses

To examine the potential differences of tumor cellular characteristics in recurrence, we compared pathological subtypes, tumor grades and cellularity across the four groups (Table 2). We examined 30 cases of PA among the 386 cases, which were chosen in cases that were treated in 2018. Consequently, tumor cellularity and stroma proportion according to the published criteria were similar between PA and RPA [19,20].

In the malignant components of CXPA and CXRPA, the most frequent subtypes were salivary duct carcinoma (*n* = 12, 40.0%), adenocarcinoma not otherwise specified (*n* = 9, 30.0%), and myoepithelial carcinoma (*n* = 5, 16.7%). Among them, high-grade tumors accounted for 30–50% of all carcinomas. Some of the salivary duct carcinomas (3 out of 12) were non-invasive carcinomas. Particularly, all myoepithelial carcinomas were low-grade (*n* = 5) in CXPA and CXRPA, which was consistent with the previous report [21]. Myoepithelial carcinoma is known to be a low-grade cancer when it arises in a pleomorphic adenoma and high grade when it is de novo [21]. The features of the carcinoma subtypes of CXPA and CXRPA were comparable.

### 3.3. Longitudinal Time–Event Sequences of Recurrence or Malignant Transformation

We focused on the time–event sequences of recurrence or malignant transformation in CXPA, RPA and CXRPA (Table 3). Disease onset was determined based on medical documents or patient history. Duration of disease, which was defined by the time from disease onset to its diagnosis, was not different among the four groups. The time intervals for benign recurrences in RPA and CXRPA (before malignant transformation) were also similar.

We next calculated the time intervals of malignant transformation (from onset of disease to diagnosis of malignancy) in CXPA and CXRPA. In CXPA, this interval was approximately 1–2 years. In contrast, the duration from the onset of underlying PA to malignancy was more than 15–20 years in CXRPA (*p* = 0.003). The time interval from benign recurrence to malignancy was approximately 11–13 years. In addition, disease duration from onset to malignancy in CXRPA was significantly longer than the time interval of benign recurrence in RPA. These results suggest that a long incubation time seems to be necessary for malignant transformation of CXRPA from RPA. 

**Figure 2:**
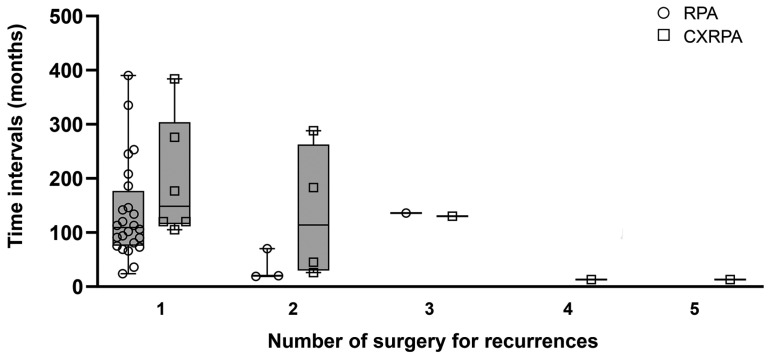
Recurrence intervals according to the number of recurrences (surgery) in recurrent pleomorphic adenoma and carcinoma ex recurrent pleomorphic adenoma. Recurrence intervals according to the number of recurrence were not significantly different between RPA and CXRPA. RPA, recurrent pleomorphic adenoma; CXRPA, carcinoma ex recurrent pleomorphic adenoma; Box plot, median, interquartile range, total range.

We then investigated whether recurrence or repeated surgical procedures can promote malignant transformation from RPA. If true, time intervals between recurrences may be shorter in CXRPA than those in RPA. Without comparison to RPA, time intervals of recurrence decreased gradually in CXRPA as the number of recurrences increased, and repeated recurrences seemed to promote malignant transformation (malignant recurrences) (Figure 2). However, the time intervals between recurrences did not differ significantly in CXRPA and RPA. 

The malignancy rate of the first recurrence (24 in RPA and 6 in CXRPA) was 13.3% (*n* = 4). The number of malignant transformations in the second recurrence (3 in RPA and 2 in CXRPA) was only one (1 of 5, 20.0%), excluding two cancer recurrences in CXRPA. Therefore, the malignancy rates of the first and second recurrences were not significantly different (*p* = 0.561). Malignancy occurrence rates for the third, fourth, and fifth recurrences were not calculated because there were only one or two cases of each.

### 3.4. Overall and Adjusted Rates of Malignant Transformation in Underlying PA

**Figure 3:**
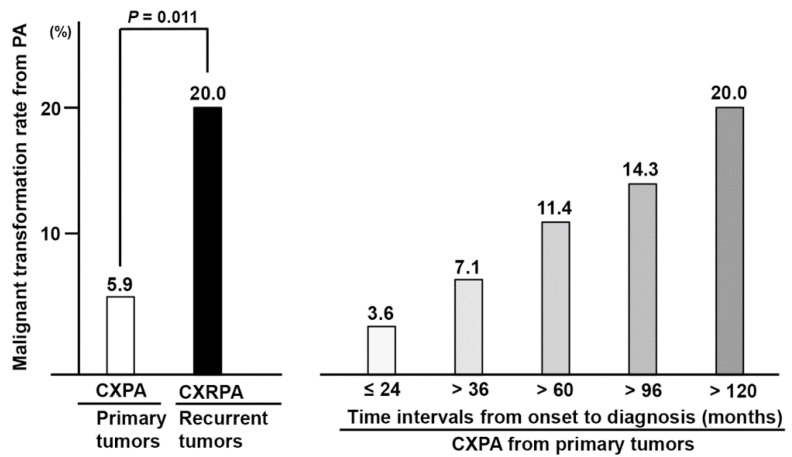
Overall incidence of malignant transformation arising from benign pleomorphic adenoma in our series. The malignancy rate (carcinoma ex pleomorphic adenoma) among the primary tumors was recalculated according to the incubation time (onset to diagnosis) intervals. CXPA, carcinoma ex pleomorphic adenoma; CXRPA, carcinoma ex recurrent pleomorphic adenoma.

The overall rates of malignant transformation from primary tumors and recurrent tumors have been frequently used as supporting evidence of the malignancy-promoting effect of recurrence or repeated surgeries [10,11,12,13]. Therefore, we calculated the overall incidence of malignant transformation in our series, which was 5.9% in primary tumor cases and 20.0% in recurrent cases (*p* = 0.011) (Figure 3). However, this calculation did not consider the time intervals from disease onset to diagnosis (incubation time). As mentioned previously, the time interval from the diagnosis of a benign tumor to malignancy was approximately 11–13 years in CXRPA (Table 3). Thus, we recalculated the rate of malignant transformation among the primary tumors, according to the various duration of the so-called incubation time. Interestingly, the rate increased up to 11.4% in cases with an incubation time of more than 5 years, and further to 20.0% in cases with an incubation time of more than 10 years (Figure 3). In summary, the rate of malignant transformation was not significantly different between primary tumors and recurrent tumors, when we adjusted it for the time interval from onset to diagnosis.

## 4. Discussion

It is important to recognize that the observed incidence rates (5.9–20.0%) of malignant transformation in our series did not reflect the actual population incidence. Our hospital is a nationwide referral center for salivary gland cancers. Therefore, severe or complicated cases (for example, recurrent or malignant disease) tend to be referred to our center. For this reason, our cancer incidence rates among benign PA or RPA are likely overestimated compared with the population (Figure 3). There is a large gap between our results and the reported rates of malignant transformation (less than 0.5% of malignant transformation for primary tumor, and 3–7% for recurrent PA) [1,13,14].

Many prior studies have investigated the malignant transformation of PA [4,22,23,24,25,26,27]. However, most of these studies explored the chronological evolution or biological changes of PA to CXPA without adequate comparison groups. In contrast, our study included cases of PA, RPA and CXPA as control groups to CXRPA. These comparisons enabled us to identify the relative contribution of several factors on the malignant transformation of PA.

A basic assumption of this paper is that benign recurrence of PA is a consequence of microscopic residual disease that is mainly due to incomplete surgical resection or a contaminated surgical field [7,28,29]. Meanwhile, malignant transformation may be induced by tumor intrinsic factor, which is a time-dependent process [7]. If these hypotheses are valid, then the long incubation time of residual disease in RPA may lead to malignant transformation in CXRPA. Concordantly, we found that disease duration from onset to malignancy in CXRPA was significantly longer than the time interval of benign recurrence in RPA. 

Clinical observations revealed that recurrence intervals decreased gradually and malignancy rates increased with more recurrences of RPA and CXRPA [28]. This finding has led to the hypothesis that recurrences or repeated surgeries may promote malignant transformation of PA [28]. In our series, the time intervals between recurrences did not differ significantly in CXRPA and RPA (Figure 2), which was an adequate control group to CXRPA. Furthermore, the malignancy rates in cases of first and second recurrence were similar in our series. In short, recurrences or repeated surgeries did not significantly impact malignant transformation of PA, which is concordant with the previous results [14].

Next, we re-examined the overall rate of malignant transformation among primary tumors, according to incubation time duration. Interestingly, the rates of malignant transformation in primary tumors gradually increased with incubation times of more than 5 years. There was a further increase in cases with incubation times of more than 10 years, which comes close to the malignancy rate of recurrent tumors. 

Our findings are contradictory to those of previous studies [10,11,12,13], in which authors argued that recurrence is a risk factor for malignant transformation in PA. However, most prior studies did not compare it with recurrences in RPA without malignant transformation. They also did not consider the duration of disease in CXRPA in comparison to that of RPA and others.

It is also worth noting that the time intervals from disease onset to malignancy in CXPA and CXRPA were significantly different in our series (longer in CXRPA than in CXPA) (Table 3). However, time intervals (onset time point) or incubation time of CXPA may not have been estimated accurately by patients, in comparison to cases of repeated recurrences (documented). Another explanation is that a subset of primary CXPA may be derived from time-independent tumor factors. In CXPA with luminal differentiation, the malignant transformation of ductal epithelial cells may follow a stepwise sequence (maybe due to the accumulation of genetic instabilities) and manifest as carcinoma in situ, intracapsular carcinoma, and extracapsular invasive carcinoma, sequentially [30,31,32]. However, this concept of carcinoma in situ cannot be applicable to CXPA with non-luminal differentiation [4]. Therefore, malignant transformation seems to be a complex process involving tumor biological characteristics. 

Despite our comparative analyses, our study has several limitations, so caution must be taken in interpreting our results. We collected retrospective clinical data from a small number of cases, which largely depended on patient’s history (including the onset time point of disease). A prospective long-term cohort study is needed to confirm our findings, although it would be difficult to conduct such a study for these rare diseases. We also did not investigate the molecular or biological markers that reflect tumor characteristics and the potential role of radiation treatment. Nevertheless, our findings offer important insights to clinicians and researchers regarding malignant transformation of PA. 

A previous study concluded that a more conservative approach for RPA was adequate for selected RPA cases because of the low risk of malignant transformation and the high risk of facial nerve injury [14]. In that study, authors presented MT cases from RPA (*n* = 17 in the first recurrent tumors, *n* = 6 in the second or more recurrent tumors); however, the time intervals to MT were not compared with those of benign recurrences. In contrast, we could analyze the relative time intervals to MT formation and benign recurrences by setting up the optimal control group. As a result, we found that the duration of disease from its onset to malignancy in CXRPA was significantly longer than the time interval of benign recurrence in RPA. In short, we found in this study that a long incubation time of microscopic residual disease may be a risk factor for CXRPA. Our preliminary conclusion supports complete treatment of PA or RPA, including comprehensive surgery or radiation treatment, so as to not leave residual disease behind.

## 5. Conclusions

In comparison of CXRPA with CXPA or RPA, we did not find any evidence that recurrence or repeated surgeries promote malignant transformation in PA. Rather, a long incubation time of microscopic residual disease seems to be a major factor for CXRPA development in cases of underlying RPA.

## Figures and Tables

**Table 1:** Subject characteristics.

No. Patients	PA (*n* = 386)	RPA (*n* = 24)	*p*-Value	CXPA (*n* = 24)	CXRPA (*n* = 6)	*p*-Value
Age at diagnosis (years, median, IQR)	47.0 (35.0–57.0)	38.0 (31.0–49.0)	0.044	58.5 (47.0–72.0)	50.5 (43.0–58.0)	0.139
Sex (M:F, No.,%)	149:237 (38.6:61.4)	9:15 (37.5:62.5)	0.914	19:5 (79.2:20.8)	6:0	0.229
Sites of tumor origin (No.)	PG: 386	PG: 24		PG: 24	PG: 5 Lacrimal gland: 1	
No. of surgery	*n* = 386	*n* = 28		*n* = 21	*n* = 10	
Tumor size (max. diameter) (cm, median, IQR)	2.4 (1.8–3.0)	1.5 (1.0–2.6)	0.001	2.8 (2.0–4.0)	3.2 (2.8–3.8)	0.514
Multiplicity (single vs. multiple, No.,%)	381:5 (98.7:1.3)	14:14 (50.0:50.0)	<0.001	18:3	7:3	0.223
* TNM staging				T1-2N0M0 = 13, T3-4N0M0 = 6, T3-4N(+)M0 = 2, M1 = 3	T1-2N0M0 = 4, T3-4N0M0 = 2, T3-4N(+)M0 = 3 M1 = 1	
Surgery (conservative: wide resection, No., %)	373:13 (96.6:3.4)	22:6 (78.6:21.4)	0.001	10:11 (47.6:52.4)	1:9 (10.0:90.0)	0.055
Postoperative facial weakness (>6 months) (No.,%)	10 (2.6)	7 (25.0)	<0.001	1 (4.8)	5 (50.0)	0.007
Adjuvant radiation (No., %)				15 (62.5)	6 (60.0)	>0.999

PA, pleomorphic adenoma; RPA, recurrent pleomorphic adenoma; CXPA, carcinoma ex pleomorphic adenoma; CXRPA, carcinoma ex recurrent pleomorphic adenoma; PG, parotid gland; IQR, interquartile range. * TNM staging: Tumor-Node-Metastasis staging according to the American Joint Committee on Cancer (AJCC) staging manual, 8th edition.

**Table 2:** Pathological tumor subtypes.

No. (%)	Pathology Subtypes or Carcinoma Component	Tumor Grade	Pathology Features
PA (*n* = 30)	* Type 1 = 23 (76.7) Type 2 = 4 (13.3) Type 3 = 3 (10.0) Type 4 = 0		Incomplete capsule = 0 Satellite nodule = 1 (3.3) Positive resection margin = 1 (3.3)
RPA (*n* = 24)	* Type 1 = 17 (70.8) Type 2 = 5 (20.8) Type 3 = 2 (8.3) Type 4 = 0		Incomplete capsule = 2 (8.3) Satellite nodule = 14 (58.3) Positive resection margin = 6/28 surgeries (21.4)
CXPA (*n* = 24)	Salivary duct carcinoma = 10 (41.7), (Non-invasive type = 3) Adenocarcinoma NOS = 6 (25.0), Myoepithelial carcinoma = 4 (16.7), Adenoid cystic carcinoma = 1 (4.2), Mucoepidermoid carcinoma = 1 (4.2), Unknown = 2 (8.3)	High-grade = 8 (33.3), Low-grade = 14 (58.3), Unknown = 2 (8.3)	Infiltrative border = 9 (37.5) Pushing border = 13 (54.1) Unknown = 2 (8.3)
CXRPA (*n* = 6)	Adenocarcinoma NOS = 3 (50.0), Salivary duct carcinoma = 2 (33.3), Myoepithelial carcinoma = 1 (16.7)	High-grade = 3 (50.0), Low-grade = 3 (50.0),	Infiltrative border = 5 (83.3) Unknown = 1 (16.7)

PA, pleomorphic adenoma; RPA, recurrent pleomorphic adenoma; CXPA, carcinoma ex pleomorphic adenoma; CXRPA, carcinoma ex recurrent pleomorphic adenoma; adenocarcinoma NOS, not otherwise specified. * Pleomorphic adenoma subtypes. Type 1: 30–50% of myxoid stroma, type 2: >80% of myxoid stroma, type 3: <30% of myxoid stroma and cells > 80%, type 4: <30% of myxoid stroma and uniformly differentiated epithelial cells.

**Table 3:** Time–event sequences of recurrence and malignant transformation in pleomorphic adenoma.

Time Intervals (Months, Median, IQR)	PA (*n* = 386)	RPA (*n* = 24)	CXPA (*n* = 24)	CXRPA (*n* = 6)	*p*-Value
Onset to the 1st diagnosis	12.0 (4.0–24.0)	11.0 (2.0–15.0)	8.0 (2.0–42.0)	16.5 (12.8–19.5)	NS
Onset to recurrence (benign)		109.5 (79.5–156.0) (1)		148.5 (134.3–162.8)	NS
Onset to malignant transformation			8.0 (2.0–42.0) (2)	342.0 (190.5–394.5) (3)	(1) vs. (3): *p* = 0.003 (2) vs. (3): *p* = 0.004

Timeline: onset (patient history)–diagnosis and treatment (PA or CXPA)–recurrence (RPA)–malignant transformation (CXRPA). PA, pleomorphic adenoma; RPA, recurrent pleomorphic adenoma; CXPA, carcinoma ex pleomorphic adenoma; CXRPA, carcinoma ex recurrent pleomorphic adenoma; IQR, interquartile range; NS, not significant statistically. (1), (2), (3) are necessary for *p*-value, indicating comparison groups.

## Data Availability

The data presented in this study are available on request from the corresponding authors.
